# Primary surgery versus no surgery in synchronous metastatic breast cancer: patient-reported quality-of-life outcomes of the prospective randomized multicenter ABCSG-28 Posytive Trial

**DOI:** 10.1186/s12885-020-06894-2

**Published:** 2020-05-06

**Authors:** V. Bjelic-Radisic, F. Fitzal, M. Knauer, G. Steger, D. Egle, R. Greil, P. Schrenk, M. Balic, Ch. Singer, R. Exner, L. Soelkner, Michael Gnant, on behalf of the ABCSG

**Affiliations:** 1 Breast Unit, University Hospital Wuppertal, Heusnerstraße 40, 42283 Wuppertal, Germany; 2 Department of Gynecology and Obstetrics, Medical University Graz, Graz, Austria; 3 Department of Surgery and Comprehensive Cancer Center, Medical University of Vienna, Vienna, Austria; 4 Breast Unit, Kantonsspital St. Gallen, St. Gallen, Switzerland; 5 Department of Internal Medicine, Medical University of Vienna, Vienna, Austria; 6 Department of Gynecology and Obstetrics, Medical University Innsbruck, Inssbruck, Austria; 7 Department of Internal Medicine III with Hematology, Medical Oncology, Hemostaseology, Infectious Disease, Rheumatology, Oncologic Center, Laboratory for Immunological and Molecular cancer Reseasrch, Paracelsus Medical University Salzburg, Salzburg, Austria; 8 Department of Surgery, Medical University Linz, Linz, Austria; 9 Division of Oncology, Department of Internal Medicine and Comprehensive Cancer Center, Medical University Graz, Graz, Austria; 10 Department of Gynecology and Obstetrics, Medical University Vienna, Vienna, Austria; 11 Statistics Department, Austrian Breast and Colorectal Cancer Study Group (ABCSG), Vienna, Austria

**Keywords:** breast cancer, metastatic cancer, cancer management, quality of life

## Abstract

**Background:**

The ABCSG-28 trial compared primary surgery followed by systemic therapy versus primary systemic therapy without surgery in patients with de novo stage IV BC. The present report describes QoL results of this trial.

**Methods:**

Ninety patients with primary operable MBC were randomised to surgery of the primary tumor followed by systemic therapy or to primary systemic therapy without surgery. QoL analyses covering the results at baseline, 6,12,18 and 24 months follow up of 79 (88%) patients, was assessed with the EORTC QLQ-C30 and QLQ-BR23 questionnaires.

**Results:**

There were no statistically significant differences in any of the scales of the QLQ-C30 and QLQ-BR23 questionnaires between the two groups over the time. Baseline global health status and physical functioning were predictors for OS (patients with a higher score lived longer (*p*=0.0250, *p*=0.0225; *p*=0.0355, *p*=0.0355)). Global health status, social functioning scale, breast symptoms and future perspective were predictors for longer TTPd (*p*=0.0244; *p*=0.0140, *p*=0.020; *p*=0.0438, *p*=0.0123). Patients in both arms reported significant improvement on the emotional functioning scale. Cognitive functioning decreased over time in both groups. Younger women had clinically relevant better physical and sexual functioning scores (*p*=0.039 and 0.024).

**Conclusion:**

Primary surgery does not improve nor alter QoL of patients with de novo stage IV BC. Global health status and physical functioning were predictors for OS and could be use as additional marker for prediction of OS and TTTd in patients with de novo stage IV BC.

**Trial registration:**

The trial is registered on clinicaltrial.gov (NCT01015625, date of registration:18/11/2009).

## Background

Breast cancer (BC) remains by far the most frequent type of cancer in women, with 1.7 million new cases and more than 500.000 deaths annually worldwide [1]. Despite large-scale efforts directed towards early detection, about 25% of newly diagnosed breast cancer patients have metastases at the time of diagnosis [2]. The median survival of metastatic breast cancer (MBC) patients improved significantly between 2000 and 2010 as compared to the previous decade and is expected to rise further [3]. This is particularly true for patients younger than 49 years, whose 5-year overall survival increased from 18% to 36% with an increase of median OS from 22.3 to 38.7 months. 11% of women younger than 64 years diagnosed with metastatic breast cancer between 2000 and 2004 survived longer than 10 years [3]. Treatment goals in patients with MBC are to prolong survival and preserve their quality of life (QoL) [4, 5].

It remains unclear whether patients presenting with MBC benefit from surgery [6, 7]. It is unknown whether surgery impacts the survival outcomes of these women [6–12], or whether surgery might improve QoL by eliminating the primary tumor. In 2011 the Austrian Breast and Colorectal Study Group (ABCSG) initiated a randomized trial of primary surgery versus primary systemic therapy in women with primary synchronous MBC (ABCSG 28, Primary breast operation in synchronous metastasized invasive breast cancer; Posytive Trial) [13]. This study, which was halted prematurely because of a slow accrual of patients, still demonstrated that surgery provided no benefit in overall survival (OS), time to distant metastases (TTPd), or time to locoregional metastases (TTPl) [13]. Given that surgery fails to improve survival, QoL in this population becomes an important decision tool. The present report describes the QoL results of the Posytive Trial.

## Methods

The ABCSG 28 trial (ClinicalTrials.gov NCT01015625) was a prospective, multicenter, randomized, phase III study in patients with primary MBC, the primary outcomes of which have been reported [13].

**Fig. 1:**
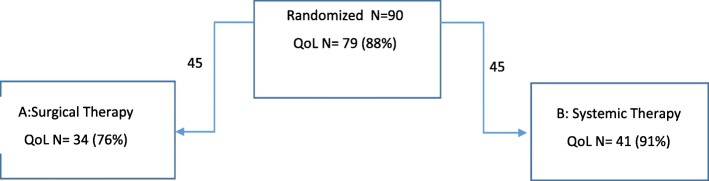
Consort diagram of the patients randomised to the ABCSG-28 Positive trial with QoL assessment

The primary aim of the study was to investigate whether upfront resection of the breast tumor followed by standard radiation and systemic therapy improved median survival compared with no surgical resection. Secondary endpoints were time to distant and locoregional progression (TTPd; TTPl) and assessment of QoL. The trial randomized patients with primary operable BC with visceral and/or non-visceral metastases (with or without biopsy of the metastases) in 15 centres in Austria between 2011 and 2015. The patients were stratified according to grading, receptor status, HER2 status, location of metastasis (visceral vs bone-only metastases),and planned first-line therapy. The trial intended to randomize 254 patients but was stopped prematurely at 4 years because of slow recruitment. At the time recruitment was stopped the trial had enrolled 90 patients, with 45 randomised into each arm [13]. The present report describes QoL results of this trial. (Consort diagram of the patients randomized to the ABCSG-28 Positive trial with QoL assessment is presented in Fig. 1.) The ABCSG 28 [13] and the present analysis of QoL data adheres to CONSORT guidelines.

The trial is listed on clinicaltrial.gov (NCT01015625) and has been approved by local ethic authorities of each centres. All patients signed informed consent.

### QoL assessment

QoL was assessed with the EORTC QLQ-C30 (Version 3.0) core questionnaire [14] and the EORTC QLQ-BR23 questionnaire for breast cancer patients [15]. Patients completed the questionnaires before randomisation and every 6 months during follow-up.

The EORTC QLQ-C30 consists of 30 items measuring global health/QoL scale, functioning scales (physical, role, emotional, cognitive, and social functioning scale) and symptoms scales/items (fatigue, nausea and emesis, pain, dyspnoea, insomnia, appetite loss, constipation, diarrhoea, financial difficulties). All scales and single items range from 0 to 100. High scores for functioning and global health/QoL scales indicate high/healthy levels of functioning/high QoL, whereas high scores for a symptom scales/items indicate a high level of symptoms/problems [14]. The 23-item EORTC QLQ BR23 contains five multi-item scales to assess body image, sexual functioning, systemic therapy side effects, arm symptoms and breast symptoms and single items to assess sexual enjoyment, future perspective and upset by hair loss. The multi-item scales and single items are divided in to two groups, namely functional scales: body image, sexual functioning, sexual enjoyment and future perspective and symptom scales/items: systemic therapy side effects, breast symptoms, arm symptoms and upset by hair loss [15]. All scales and single items range from 0 to 100. A high score for all functioning scales indicates high/healthy level of functioning/high QoL, whereas a high score for a symptom scale/items indicates a high level of symptoms/problems.

### Statistical analysis

The EORTC QLQ-C30 and EORTC QLQ BR23 scales and single items were linearly transformed to 0-100 and analysed according to the recommendations of the EORTC QoL Group [16]. Differences of at least 10 points on the scales/items were defined as the threshold for minimum of clinically significant difference [17]. All QoL analyses were based on the QoL-evaluable population i.e. patients in the intent-to-treat population with a baseline QOL assessment. Questionnaire completion rates were calculated for all patients per assessment time and per treatment arm. Completion rates were summarized by visit.

At that time only 90 patients were enrolled, 45 in each arm. Thus, the study is underpowered and needs to be interpreted in an explorative manner.

Patient characteristics between patient with and without QOL assessment were tested with Chi square / Fischer Test. The main QOL objective was to test whether Surgical Arm leads to improved QOL when compared with Systematic Therapy Arm, based on the Global health/QOL scale of the QLQ-C30. The primary analysis was performed by fitting a linear mixed model with treatment, a (linear) time effect, a time–treatment interaction as fixed effects and patient specific random effect on QoL-evaluable population. Treatment, time, treatment by time, and baseline were covariates for the model. A restricted maximum likelihood method assuming an unstructured covariance matrix was used.

Additional analyses were done by age, site of metastases, and type of primary systemic therapy (chemotherapy vs. other) as covariates. No adjustments were made for multiple comparisons.

Baseline Global health status/QoL, and physical functioning scale scores were split at the median to yield ‘good’ and ‘poor’ scores.

All analyses were conducted using Statistical Analysis System software (SAS Institute, Cary, NC) for Windows (Microsoft Corp., Redmond, WA). All P values are two-sided unless stated otherwise.

## Results

Between 2011 and 2015 90 patients were randomized at 15 centers, 45 patients into each arm. 79 (88%) patients completed QoL assessment at least at baseline. 34 (76%) patients in the surgery arm and 41 (91%) in the no-surgery arm were included in the QoL analyses. (Table 1) A total of 289 QoL questionnaires were analysed 79 (88%) at baseline and 60 (76%), 54 (73%), 38 (56%), 32 (52%), at 6, 12, 18 and 24 months, respectively. QoL analysis covered the results of the five assessment time points (baseline and 6,12,18 and 24 months’ follow-up).

**Table 1:** Demographic and clinical characteristics of patients randomized in the ABCSG 28 study, *n*= 90

Category	In Qol n(%)		no QoLn(%)		P-value
Number of patients					
	79	100.0)	11	100.0)	
Menopause Status					
Perimenopausal	1	(1.3)	.	.	1.0
Postmenopausal	69	(87.3)	9	(81.8)	
Premenopausal	9	(11.4)	2	(18.2)	
T-stage					
cT1	15	(19.0)	2	(18.2)	**0.0498**
cT2	33	(41.8)	7	(63.6)	
cT3	12	(15.2)	1	(9.1)	
cT4	16	(20.3)	.	.	
Missing	3	(3.8)	1	(9.1)	
N-stage					
cN0	18	(22.8)	2	(18.2)	0.4261
cN1	34	(43.0)	7	(63.6)	
cN2	9	(11.4)	.	.	
cN3	6	(7.6)	.	.	
Missing	12	(15.2)	2	(18.2)	
Grading					
G1	5	(6.3)	2	(18.2)	0.6282
G2	44	(55.7)	4	(36.4)	
G3	24	(30.4)	4	(36.4)	
Gx	3	(3.8)	1	(9.1)	
Missing	3	(3.8)	.	.	
HER2					
FISH amplif./IHC+++	15	(19.0)	5	(45.5)	0.4475
Negative	63	(79.7)	6	(54.5)	
Missing	1	(1.3)	.	.	
Hormone Status					
any positive	65	(82.3)	8	(72.7)	1.0
Negative	14	(17.7)	3	(27.3)	
Tumor Subtype					
Basal Type	8	(10.1)	.	.	0.8181
HER2 Type	15	(19.0)	5	(45.5)	
Luminal A	41	(51.9)	5	(45.5)	
Luminal B	11	(13.9)	1	(9.1)	
Missing	4	(5.1)	.	.	

*Legends: QoL- Quality of Life*

Except for tumour size, demographic and clinical characteristics in patients for whom QoL data were available and in those for whom they were not were similar (Table 2). Median age was 62.8 years and similar in both groups (61.7 vs 63.9).

**Table 2:** Systemic and local therapy of patients participated in the QoL study

	Arm A Surgical therapy *N*=37	Arm B No surgical therapy *N*=42	p*
	N (%)	N (%)	
First line therapy			
Any CTX no Taxane	6 (16.2)	5 (11.9)	
Any CTX with Taxane	4 (10.8)	10 (23.8)	
Endocrine therapy	27 (73.0)	27 (64.3)	0.308
Radiotehrapy			
Breast/Chest wall	9 (22.0)	2 (4.7)	0.020
Metastases	11(26.8)	8 (18.6)	0.268
Surgery			
Metastases	1 (2.4)	3 (7.0)	0.618

*Fischer exact test

CTX-Chemotherapy

**Fig. 2:**
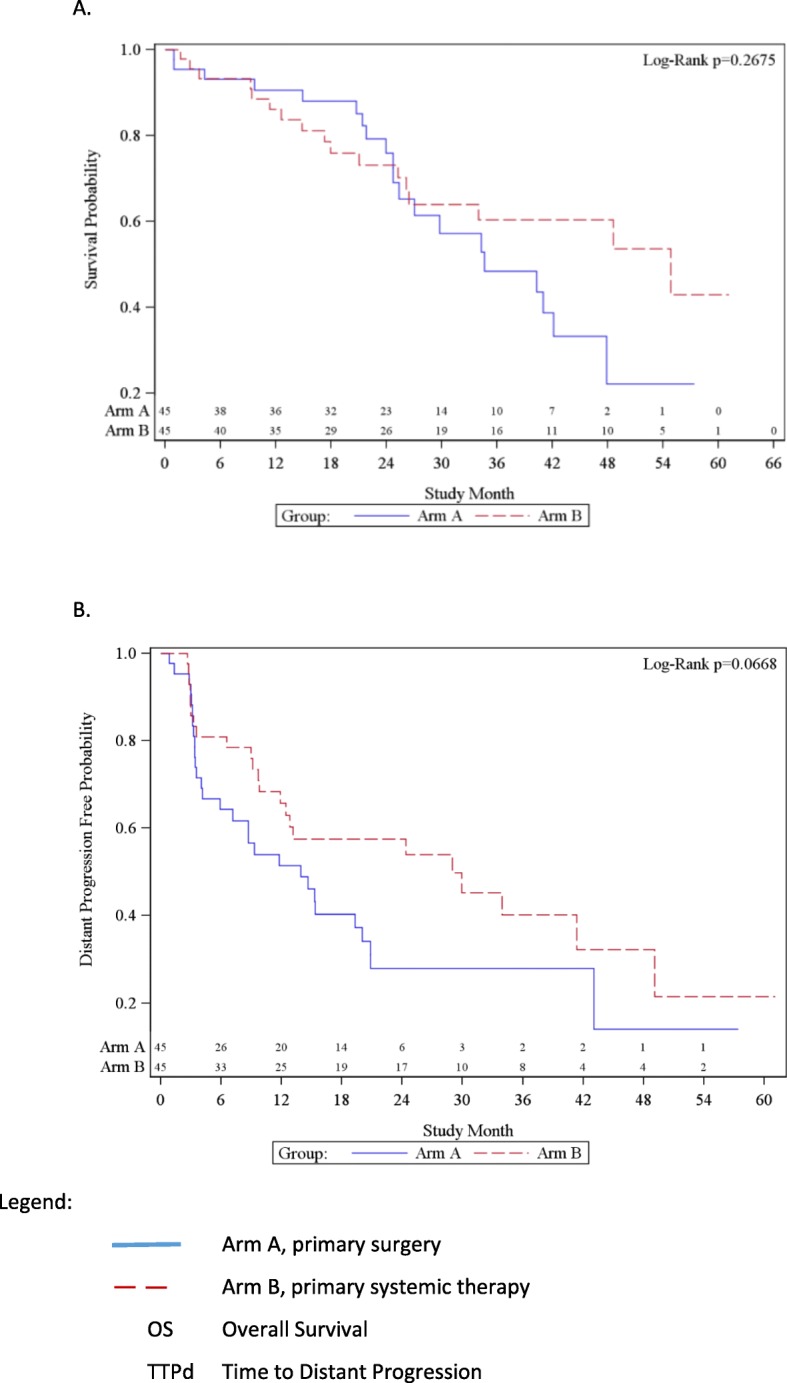
Kaplan-Meier Plot for OS and TTPd. A. OS. B. TTPd. Arm A, primary surgery.  Arm B, primary systemic therapy. OS Overall Survival. TTPd Time to Distant Progression

Survival data have been reported previousl y[13]. Surgery did not provide an OS benefit (34.6 months vs 54.8 months, *p*=0.267; HR 0.691; 95% CI 0.358–1.333 ) or TTPd and TTPl ( HR 0.598, *p*=0.0668; HR 0.933, *p*=0.882 )[13] (Fig. 2a and b).

### QoL assessment as predictor for OS and TTPd

**Fig. 3:**
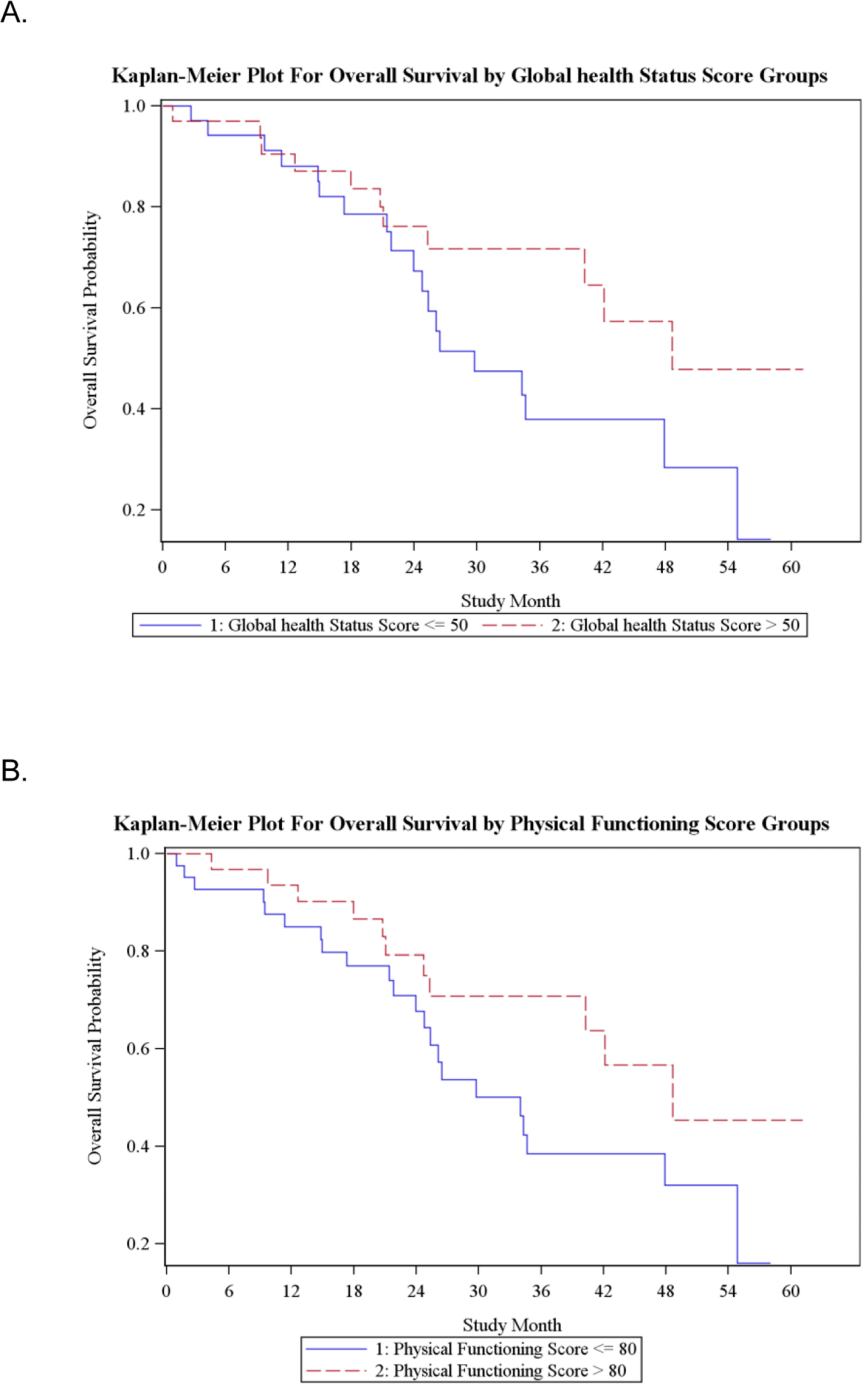
Kaplan-Meier Plot for OS by Global Health Status and Physical Fuctioning of the EORTC QLQ C30 Legends: OS - Overall Survival

In the univariate and multivariate analyses the Global health status/QoL and physical functioning scales were predictors for overall OS. Patients with a higher score of global health status/QoL and higher score of physical functioning lived longer (HR 0.984; *p*=0.0250, HR 0.984; *p*=0.0225; HR 0.988 *p*=0.0355, HR0.988; *p*=0.0355) (Fig. 3a; b, Table 3). Although not statistically significant, patients with a higher score on the scale future perspective showed a tendency to longer OS in the univariate analyses (HR 0.987; *p*=0.0510). In the univariate analyses scales Global health status/QoL and social functioning scale were a predictor for a longer TTPd (HR 0.985, *p*=0.0244; HR 0.989, *p*=0.0140)( Table 4).

**Table 3:** QoL Score as predictor for OS (univariate and multivariate analyse)

	Univariate Analyse				Multivariate Analyse			
	HR	95%-LL	95%-UL	Cox P-value	HR	95%-LL	95%-UL	Cox P-value
*Physical Functioning*	0.988	0.977	0.999	**0.0355**	1.016	0.983	1.051	0.3523
*Role Functioning*	0.993	0.984	1.001	0.0988	0.995	0.969	1.021	0.6966
*Emotional Functionin*	1.000	0.987	1.014	0.9511	1.031	1.003	1.059	**0.0293**
*Cognitive Functionin*	0.999	0.980	1.018	0.9206	0.999	0.961	1.039	0.9723
*Social Functioning*	0.996	0.986	1.006	0.4450	0.996	0.978	1.015	0.6955
*Global health status*	0.984	0.970	0.998	**0.0250**	0.960	0.932	0.990	**0.0088**
*Fatigue*	1.006	0.995	1.016	0.2874	1.002	0.973	1.031	0.9111
*Nausea / Vomiting*	1.012	0.996	1.028	0.1382	1.017	0.985	1.049	0.3077
*Pain*	1.007	0.999	1.016	0.0955	1.018	0.992	1.045	0.1828
*Dyspnoea*	1.007	0.994	1.020	0.2738	1.018	0.997	1.038	0.0882
*Insomnia*	1.003	0.992	1.014	0.6502	0.994	0.979	1.010	0.4795
*Appetite loss*	1.007	0.996	1.017	0.2027	0.996	0.967	1.026	0.7874
*Constipation*	1.002	0.990	1.013	0.7756	0.986	0.966	1.005	0.1494
*Diarrhoea*	0.994	0.974	1.014	0.5714	0.997	0.971	1.024	0.8285
*Financial Problems*	0.990	0.971	1.009	0.2845	0.977	0.950	1.004	0.0922
*BR Body image*	0.992	0.978	1.006	0.2724	0.997	0.978	1.016	0.7507
*BR Sexual functioning*	0.993	0.976	1.009	0.3856	0.995	0.976	1.014	0.5827
*BR Future perspective*	0.987	0.975	1.000	**0.0510**	0.990	0.974	1.007	0.2678
*BR Systemic therapy*	1.013	0.998	1.029	0.0888	1.008	0.977	1.040	0.6281
*BR Breast symptoms*	1.005	0.989	1.022	0.5249	1.007	0.986	1.027	0.5268
*BR Arm symptoms*	1.008	0.990	1.027	0.3686	0.996	0.973	1.020	0.7320
*BR Hair loss*	0.988	0.977	0.999	**0.0355**	0.982	0.931	1.036	0.5133
*BR Sexual enjoyment*	0.993	0.984	1.001	0.0988	0.997	0.978	1.016	0.7507

Legends: *OS Overall Survival*, *HR Hazard ratio*

**Table 4:** QoL Score as predictor for TTPd (univariate and multivariate analyse)

	Univariate Analyse				Multivariate Analyse			
	HR	95%-LL	95%-UL	Cox P-value	HR	95%-LL	95%-UL	Cox P-value
*Physical Functioning*	0.995	0.985	1.006	0.3855	1.006	0.978	1.034	0.6704
*Role Functioning*	0.995	0.987	1.004	0.2671	1.010	0.988	1.034	0.3741
*Emotional Functioning*	0.999	0.986	1.011	0.8220	1.018	0.996	1.041	0.1006
*Cognitive Functioning*	0.993	0.976	1.009	0.3852	1.005	0.979	1.031	0.7245
*Social Functioning*	0.989	0.979	0.998	**0.0140**	0.986	0.969	1.003	0.1057
*Global health status*	0.985	0.973	0.998	**0.0244**	0.983	0.961	1.005	0.1313
*Fatigue*	1.003	0.993	1.013	0.6083	1.011	0.988	1.035	0.3465
*Nausea / Vomiting*	1.010	0.994	1.026	0.2174	1.009	0.978	1.040	0.5811
*Pain*	1.006	0.998	1.015	0.1443	1.015	0.993	1.037	0.1797
*Dyspnoea*	1.007	0.996	1.018	0.1908	1.015	0.998	1.032	0.0829
*Insomnia*	1.000	0.991	1.009	0.9839	0.999	0.986	1.011	0.8211
*Appetite loss*	1.000	0.990	1.010	0.9466	0.991	0.969	1.013	0.4189
*Constipation*	0.998	0.987	1.009	0.7022	0.986	0.969	1.003	0.0992
*Diarrhoea*	0.998	0.983	1.013	0.8056	0.999	0.979	1.018	0.8917
*Financial Problems*	1.005	0.993	1.016	0.4073	0.998	0.984	1.013	0.8026
*BR Body image*	0.999	0.985	1.013	0.8986	1.001	0.985	1.017	0.8999
*BR Sexual functioning*	0.992	0.979	1.006	0.2574	1.001	0.986	1.015	0.9242
*BR Future perspective*	0.988	0.978	0.999	**0.0250**	0.982	0.968	0.996	**0.0123**
*BR Systemic therapy*	1.000	0.984	1.017	0.9664	1.010	0.984	1.036	0.4762
*BR Breast symptoms*	1.008	0.994	1.023	0.2611	1.021	1.001	1.041	**0.0438**
*BR Arm symptoms*	0.993	0.973	1.012	0.4568	0.994	0.973	1.017	0.6238
*BR Hair loss*	0.952	0.899	1.010	0.1014	0.933	0.872	0.998	
*BR Sexual enjoyment*	1.010	0.984	1.038	0.4525	1.001	0.985	1.017	0.8999

*Legends: TTPd- time to distant progression; QoL: Quality of life*

In the univariate and multivariate analyses, the scale future perspective was a predictor for longer TTPd (HR 0.988, *p*=0.020; HR 0.982, *p*=0.0123) (Table 4). In the multivariate analyses scale breast symptoms was a predictor for TTPd (HR 0.933, *p*=0.0438)( Table 4).

### QoL assessment by therapy arm

**Fig. 4:**
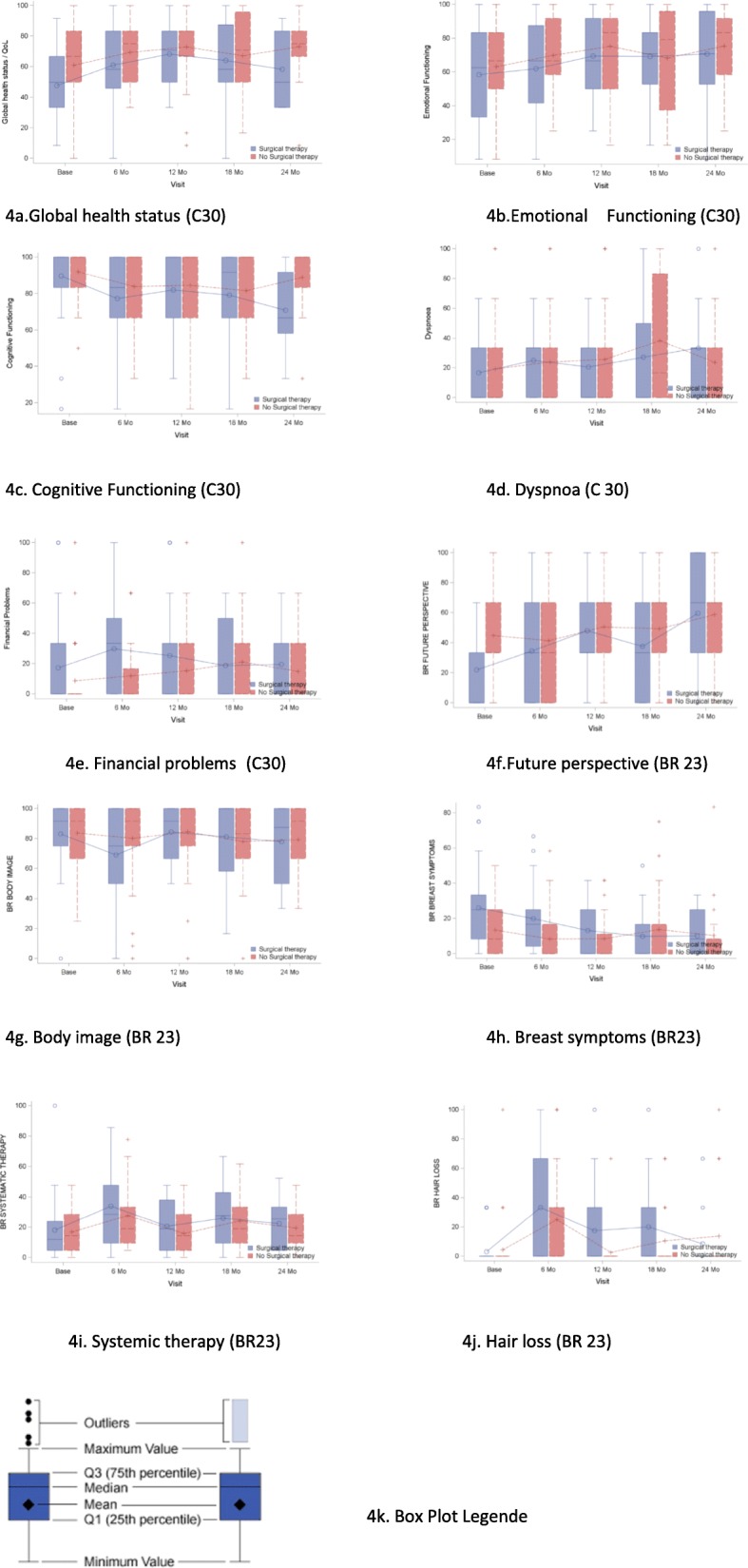
QoL scales (EORTC QLQ C30 and EORTC QLQ BR 23) by therapy arm with statistical significant changes over the time. Legende.4a Global health status (C30) p=0.003; 4b. Emotional Functioning (C30) p =0.013; 4c. Cognitive Functioning (C30) p=0.006; 4d. Dyspnoea (C 30) p=0.026; 4e. Financial problems (C30) p=0.031; 4f.Future perspective (BR 23) p=0.009; 4g. Body image (BR 23) p=0.018; 4h. Breast symptoms (BR23). p=0.006; 2i. Systemic therapy (BR23) p<0.0001; 4j. Hair loss (BR 23) p<0.0001; 4k. Box Plot Legende:  Mean value therapy arm A -Surgical therapy; + mean value thearpy arm B- no surgical therapy

Details of the systemic and local therapy in the surgical and no surgical arm are listed in the Table 2. There were no statistically significant differences in any of the scales of the QLQ-C30 and QLQ-BR23 questionnaires between the two groups over the time. (Table 5) Figure 4 presented QoL scale with statistically significant change (improvement or worsening) over the time in both groups.

**Table 5:** QoL assessment (EORTC QLQ C30 and EORTC QLQ BR 23) by therapy arm and assessment time (Mean scores and standard errors)

		baseline		6 mo		12 mo		18 mo		24 mo			
	Ref	Arm A	Arm B	Arm A	Arm B	Arm A	Arm B	Arm A	Arm B	Arm A	Arm B	p-value^b^	p-value^c^
Physical Functioning	81.6 (18.7)	69.2 (4.8)	72.8 (4.5)	71.1 (4.8)	75.4 (4.7)	70.1 (4.8)	73.1 (4.7)	65.7 (6.1)	70.1 (5.8)	62.4 (5.9)	70.1 (5.4)	0.4585	0.3822
Role Functioning	67.4 (31.1)	65.5 (6.3)	69.2 (5.7)	62.3 (6.4)	65.9 (6.4)	65.8 (6.0)	67.9 (5.9)	58.7 (7.8)	69.7 (7.3)	56.4 (8.8)	68.8 (7.9)	0.3676	0.8800
Emotional Functioning	65.9 (24.6)	58.1 (4.3)	62.3 (4.1)	62.5 (5.0)	71.4 (5.0)	68.2 (4.5)	73.6 (4.4)	65.2 (5.5)	69.6 (5.2)	69.5 (6.3)	70.6 (5.4)	0.4128	**0.0127**
Cognitive Functioning	80.5 (23.2)	89.3 (2.9)	91.6 (2.8)	79.5 (4.1)	83.5 (4.1)	82.0 (4.3)	82.5 (4.3)	78.6 (5.1)	81.5 (4.9)	74.3 (5.2)	83.3 (4.5)	0.4537	**0.0058**
Social Functioning	74.2 (28.4)	65.9 (5.6)	76.1 (5.3)	68.6 (5.7)	78.6 (5.8)	76.9 (5.7)	78.2 (5.6)	70.9 (7.4)	72.1 (6.7)	74.3 (6.2)	77.2 (5.3)	0.5507	0.3784
Global health status/QoL	60.2 (25.5)	**47.8 (4.3)**	**61.6 (4.2)**	61.4 (4.5)	68.7 (4.5)	66.0 (4.2)	69.7 (4.2)	63.5 (6.3)	68.7 (5.8)	61.4 (5.9)	71.2 (4.9)	0.2194	**0.0032**
Fatigue	36.3 (27.0)	39.3 (5.1)	34.0 (4.7)	43.2 (5.7)	35.2 (5.6)	41.8 (5.6)	37.4 (5.4)	40.8 (6.4)	32.0 (5.9)	47.5 (5.7)	31.2 (5.1)	0.1717	0.6495
Nausea/Vomiting	10.3 (19.7)	10.5 (2.9)	5.6 (2.7)	9.3 (2.6)	4.9 (2.6)	11.9 (3.7)	6.9 (3.7)	8.0 (3.4)	6.0 (3.1)	18.8 (6.0)	9.4 (5.4)	0.1930	0.2564
Pain	30.9 (29.6)	37.9 (6.2)	32.2 (5.7)	30.9 (5.5)	22.7 (5.5)	25.7 (5.1)	24.1 (5.1)	28.6 (6.4)	28.3 (6.0)	32.4 (6.6)	26.2 (6.0)	0.5478	0.2441
Dyspnoea	20.4 (28.2)	15.7 (4.6)	19.1 (4.3)	22.6 (5.3)	24.5 (5.2)	21.9 (5.8)	28.3 (5.7)	34.9 (8.8)	36.7 (8.2)	37.1 (6.9)	26.5 (6.4)	0.9850	0.0257
Insomnia	33.1 (32.6)	39.5 (5.6)	35.4 (5.3)	36.9 (5.7)	22.1 (5.7)	32.8 (6.1)	30.5 (6.0)	24.5 (7.0)	37.1 (6.5)	32.0 (7.1)	31.0 (6.3)	0.8455	0.5016
Appetite loss	21.7 (31.0)	23.3 (5.7)	20.9 (5.2)	22.8 (5.7)	11.7 (5.7)	20.2 (5.2)	14.9 (5.2)	17.3 (5.0)	11.6 (4.5)	23.3 (5.7)	13.1 (5.0)	0.1297	0.5374
Constipation	19.2 (28.8)	18.9 (5.1)	21.0 (4.9)	25.3 (6.4)	19.9 (6.4)	19.4 (5.5)	21.9 (5.5)	11.3 (6.3)	21.3 (5.9)	18.3 (7.9)	22.6 (6.8)	0.6710	0.5871
Diarrhoea	5.8 (15.2)	3.9 (3.2)	8.7 (3.1)	10.1 (4.8)	9.0 (4.6)	12.9 (3.6)	4.9 (3.5)	13.8 (4.9)	6.7 (4.7)	7.7 (3.3)	4.4 (3.1)	0.2765	0.2127
Financial Problems		16.3 (4.4)	9.4 (4.1)	27.4 (5.2)	11.2 (5.1)	27.1 (5.9)	18.8 (5.7)	27.3 (6.5)	23.8 (6.2)	19.5 (5.3)	18.3 (4.9)	0.2845	**0.0308**
Body image	81.9 (22.6)	83.2 (3.7)	83.2 (3.5)	71.2 (5.4)	78.1 (5.2)	82.9 (4.2)	81.7 (4.0)	78.5 (5.3)	79.5 (4.8)	73.1 (5.6)	74.9 (5.1)	0.7139	**0.0178**
Sexual functioning	19.2 (23.2)	12.6 (4.4)	18.5 (4.2)	17.4 (4.5)	17.2 (4.4)	15.6 (5.7)	24.3 (5.4)	18.6 (6.2)	22.5 (5.2)	12.6 (6.0)	24.6 (5.2)	0.3300	0.7514
Future perspective	47.6 (34.1)	21.4 (5.4)	45.0 (5.0)	33.8 (6.1)	42.5 (5.9)	46.0 (6.5)	49.9 (6.2)	31.0 (7.7)	49.5 (6.9)	52.2 (8.2)	54.8 (7.5)	0.2336	**0.0093**
Systematic therapy	15.8 (14.3)	18.4 (3.0)	17.0 (2.7)	31.9 (4.1)	27.9 (4.1)	21.4 (3.0)	17.8 (2.9)	25.1 (4.0)	23.9 (3.7)	23.3 (3.5)	21.9 (3.2)	0.5214	**<.0001**
Breast symptoms	17.6 (16.7)	26.7 (3.4)	14.1 (3.2)	20.1 (3.0)	8.8 (3.0)	13.7 (2.6)	9.9 (2.6)	10.4 (3.9)	12.0 (3.6)	7.3 (3.9)	10.6 (3.6)	0.4559	**0.0056**
Arm symptoms	21.0 (21.1)	18.1 (3.2)	14.2 (3.0)	28.3 (4.3)	14.5 (4.2)	23.7 (4.0)	15.1 (3.8)	29.1 (5.1)	19.2 (4.8)	18.3 (4.7)	15.8 (4.4)	0.0949	0.0575
Hair loss	5.3 (19.3)	2.6 (2.6)	4.6 (2.4)	32.5 (7.1)	27.6 (7.1)	16.3 (4.8)	-0.4 (4.3)	18.3 (5.5)	8.0 (5.0)	9.9 (7.6)	13.0 (6.8)	0.2203	**<.0001**

*Legends: Arm A: primary surgery followed by systemic therapy ; Arm B: primary systemic therapy without surgery; Ref: reference data*

*a Estimates for themean scores estimated via the linearmixedmodeling expressed in absolute score points of the scale. Higher values for the symptom scales (Diarrhea, Loss of appetite, Nausea/vomiting, Fatigue) represent worse level of symptoms. Higher values for the global health/Quality of Life scale represents a better level of functioning*

*b: p-value belongs to the comparison between therapy arms; c p-value belongs to test for time effect*

### QLQ C30

#### Global Health Status/QoL

At baseline, clinically relevant (>10 points differences) differences favouring the no-surgery arm were found in the Global Health Status/QoL scale (mean, 47.8 vs 61.6) (Table 5). These preferences disappeared at the first follow-up (6 months) and were not seen at further time points. Over time (up to 24 months follow up) patients in both arms had a clinically relevant and statistically significant improvement on the scale global health status (*p*=0.003) (Fig. 4a)

#### Functional scales of the QLQ-C30

There were no statistically significant differences in any of the five functional scales of the QLQ-C30 [physical, role, emotional, cognitive and social functioning] at baseline, as well as over time. Patients reported significant improvement on the scale emotional functioning in both arms over time (Fig. 4b). In the surgical arm this improvement was clinically relevant. Cognitive functioning decreased over time in both groups, clinically relevant and statistically significant in the primary surgery arm and statistically significant without clinical relevance in the non-surgery arm (Fig. 4c).

#### Symptom scales/Items of the EORTC QLQ-C30

The mean scores of symptoms scales/items at baseline and during follow-up remained on the lower part of the 0-100 scale. Statistically significant worsening was found on the scale dyspnoea (*p*=0.025), but this difference was without clinical relevance in both arms (Fig. 4d).

Over time patients reported more financial problems in both arms (Fig. 4e).

#### Functional scales of the QLQ-BR23

In both arms statistically significant and clinically relevant improvement was seen over time on the scale future perspective (*p*=0.009) (Fig. 4f). In contrast, patients in both arms reported worsening symptoms on the body image scale, clinically relevant in the surgery arm (*p*=0.017, Fig. 4g). At baseline women in the non-surgery arm reported a statistically significant and clinically relevant better mean score in the functional scale future perspective (mean 45.0 vs 21.4). In the following visits there were no differences in any of the functional scales between two arms (Table 5).

#### QLQ-BR23 symptoms scales

In both arms, statistically significant and clinically relevant improvement was seen over time on the breast symptoms scale (*p*=0.006, Fig. 4h). Symptom worsening was found on the scales symptoms of the systemic therapy and hair loss, but these differences were without clinical relevance in both arms. (*p*<0.001, Fig. 4i, j)

#### QoL assessment by age

The median age of our study population was 64 y (range 23y-85y). 64.5% of women were older than 60 years and only 14% were premenopausal. We compared women <60 and ≥60 years to assess a possible impact of age on QoL. There were no differences in the functional or symptomatic scales of the QLQ-C30 and QLQ –BR 23 between the two groups of women except in physical functioning scale (EORTC –QLQC30) and sexual functioning scale (EORTC BR 23). As expected, younger women had a statistically significant and clinical relevant better mean score of the physical functioning scale (*p*=0.039) and sexual functioning score (*p*=0.024) (Table 6).

**Table 6:** QoL assessment (EORTC QLQ C30 and EORTC QLQ BR 23) by age and assessment time (Mean scores and standard errors)

	*Baseline*		*6 Mo*		*12 Mo*		*18 Mo*		*24 Mo*		
*QOL domain*	*<60*	*>=60*	*<60*	*>=60*	*<60*	*>=60*	*<60*	*>=60*	*<60*	*>=60*	*p-Value*^*b*^
Physical Functioning	75.9 (5.5)	68.5 (4.0)	78.6 (5.7)	70.5 (4.1)	79.9 (5.7)	67.8 (4.0)	79.6 (7.0)	62.8 (4.9)	80.0 (6.8)	61.0 (4.6)	**0.0390**
Role Functioning	71.7 (7.1)	65.3 (5.2)	64.7 (7.8)	63.9 (5.6)	73.8 (7.4)	64.1 (5.0)	77.6 (8.9)	58.4 (6.2)	71.6 (10.5)	59.2 (7.1)	0.2148
Emotional Functioning	58.8 (5.0)	61.1 (3.7)	60.0 (6.0)	71.0 (4.4)	73.4 (5.5)	70.4 (3.8)	74.2 (6.2)	64.6 (4.4)	71.1 (7.3)	69.6 (4.9)	0.9035
Cognitive Functioning	87.7 (3.3)	92.0 (2.5)	77.6 (4.9)	83.8 (3.5)	83.9 (5.3)	82.0 (3.6)	85.2 (5.8)	77.9 (4.1)	86.8 (6.0)	76.6 (4.0)	0.5600
Social Functioning	65.0 (6.5)	74.6 (4.8)	67.2 (7.0)	77.0 (5.0)	82.2 (7.1)	76.2 (4.7)	75.8 (8.6)	69.8 (5.9)	80.9 (7.2)	73.5 (4.8)	0.7279
Global health status / QoL	57.3 (5.2)	53.9 (3.8)	60.3 (5.4)	67.7 (4.0)	73.5 (5.3)	65.8 (3.5)	78.0 (7.0)	60.4 (4.9)	69.5 (6.8)	66.3 (4.7)	0.3342
Fatigue	30.8 (5.8)	39.8 (4.3)	37.4 (6.8)	39.9 (5.0)	30.6 (6.6)	43.5 (4.6)	23.6 (7.1)	42.2 (5.0)	31.1 (7.1)	42.0 (4.8)	0.1158
Nausea / Vomiting	10.7 (3.4)	6.3 (2.5)	10.0 (3.1)	5.4 (2.2)	7.0 (4.7)	10.7 (3.1)	9.8 (4.0)	5.4 (2.8)	21.9 (7.5)	11.3 (5.1)	0.3712
Pain	32.5 (7.2)	36.2 (5.2)	29.8 (6.7)	25.4 (4.8)	30.0 (6.4)	22.7 (4.4)	27.4 (7.6)	29.5 (5.2)	33.3 (8.0)	27.7 (5.4)	0.6032
Dyspnoea	10.8 (5.3)	20.8 (3.9)	22.9 (6.3)	23.7 (4.7)	25.9 (7.4)	25.5 (5.0)	22.6 (10.0)	42.8 (6.9)	37.3 (8.3)	28.8 (5.6)	0.6989
Insomnia	34.0 (6.6)	39.2 (4.7)	38.1 (6.9)	24.5 (5.1)	32.1 (7.5)	31.3 (5.1)	21.6 (7.9)	35.9 (5.5)	27.0 (8.5)	33.2 (5.6)	0.8300
Appetite loss	18.6 (6.5)	24.0 (4.8)	17.2 (7.0)	17.2 (5.1)	17.2 (6.6)	17.7 (4.5)	8.8 (5.7)	17.0 (4.0)	15.7 (7.1)	18.6 (4.6)	0.6148
Constipation	11.4 (5.8)	24.5 (4.3)	24.7 (7.7)	21.4 (5.6)	19.6 (7.1)	21.6 (4.7)	9.5 (7.5)	20.7 (5.2)	10.8 (9.1)	24.7 (6.1)	0.4017
Diarhoea	9.6 (3.8)	4.7 (2.8)	15.3 (5.5)	6.5 (4.1)	8.6 (4.7)	9.3 (3.0)	13.4 (6.1)	8.9 (4.2)	9.5 (4.1)	4.6 (2.7)	0.3784
Financial Problems	14.7 (5.1)	11.4 (3.8)	30.4 (6.3)	13.5 (4.6)	29.7 (7.3)	19.1 (4.8)	34.4 (7.6)	20.8 (5.4)	24.7 (6.3)	16.5 (4.3)	0.0842
Body image	82.7 (4.2)	83.4 (3.2)	70.7 (6.3)	77.1 (4.8)	78.0 (5.0)	84.9 (3.5)	82.0 (5.9)	77.6 (4.3)	77.4 (6.6)	72.9 (4.5)	0.8575
Sexual functioning	22.2 (5.0)	12.3 (3.7)	23.1 (5.0)	13.8 (4.0)	30.8 (6.7)	15.1 (4.7)	32.6 (6.4)	13.8 (4.8)	28.7 (6.9)	14.5 (4.8)	**0.0240**
Future perspective	39.9 (6.3)	30.4 (4.9)	32.3 (7.2)	41.4 (5.3)	46.1 (8.0)	48.8 (5.4)	44.2 (8.8)	40.0 (6.4)	49.7 (10.2)	54.9 (6.6)	0.6710
Systematic therapy	16.1 (3.2)	18.7 (2.5)	33.8 (4.9)	27.9 (3.6)	20.2 (3.7)	19.2 (2.6)	20.9 (4.6)	26.6 (3.2)	25.6 (4.3)	21.6 (2.8)	0.7534
Breast symptoms	20.9 (4.1)	20.0 (3.1)	20.3 (3.8)	11.5 (2.7)	15.9 (3.2)	9.8 (2.2)	14.0 (4.6)	10.2 (3.3)	14.6 (4.8)	6.8 (3.1)	0.0711
Arm symptoms	13.7 (3.5)	17.5 (2.7)	22.9 (5.3)	20.5 (3.8)	23.3 (4.9)	17.4 (3.4)	23.1 (6.1)	24.7 (4.3)	15.9 (5.8)	17.9 (3.8)	0.8310
Hair loss	2.7 (2.9)	4.3 (2.3)	22.0 (8.4)	34.8 (6.2)	10.9 (6.2)	5.3 (4.1)	8.6 (6.7)	15.0 (4.5)	3.0 (8.8)	16.5 (6.1)	0.2799

*Legends: QoL Quality of life*

^*a*^*Estimates for themean scores estimated via the linearmixedmodeling expressed in absolute score points of the scale. Higher values for the symptomscales (Diarrhea, Loss of appetite, Nausea/vomiting, Fatigue) represent aworse level of symptoms. Higher values for the global health/Quality of Life scale represents a better level of functioning*

^*b*^*p-value belongs to the comparison between age groups*

#### QoL assessement by type of systemic therapy (chemotherapy vs. other, with or without surgery)

Overall, 79 women completed baseline QoL assessment and received chemotherapy (CTX) (*N*=25) or endocrine therapy (*N*= 54) as first-line systemic therapy. Women who received CTX reported baseline clinically better mean score on the scale physical functioning of the EORTC QLQC30 (Table 8). Over time those patients had statistically significant more diarrhoea (*p*=0.0014) (Table 7).

**Table 7:** QoL assessment (EORTC QLQ C30 and EORTC QLQ BR 23) by choice of first systemic therapy (Mean scores and standard errors)

	*Baseline*		*6 Mo*		*12 Mo*		*18 Mo*		*24 Mo*		
*QOL domain*	*1*^*st*^*line chemo*	*1*^*st*^*line other*	*1*^*st*^*line chemo*	*1*^*st*^*line other*	*1*^*st*^*line chemo*	*1*^*st*^*line other*	*1*^*st*^*line chemo*	*1*^*st*^*line other*	*1*^*st*^*line chemo*	*1*^*st*^*line other*	*p-Value*^*b*^
Physical Functioning	79.3 (5.4)	66.8 (3.9)	69.8 (5.8)	74.4 (4.1)	70.0 (5.8)	72.3 (4.1)	70.6 (7.1)	66.4 (5.0)	77.2 (6.8)	61.6 (4.6)	0.6359
Role Functioning	72.3 (7.2)	65.0 (5.2)	60.3 (7.9)	65.5 (5.5)	67.5 (7.2)	66.7 (5.1)	69.9 (9.1)	62.2 (6.4)	76.8 (10.0)	57.3 (6.7)	0.4942
Emotional Functioning	53.0 (5.1)	63.9 (3.6)	57.5 (6.2)	71.3 (4.2)	68.9 (5.5)	72.5 (3.8)	68.0 (6.5)	68.1 (4.6)	79.1 (6.8)	66.8 (4.6)	0.8325
Cognitive Functioning	87.0 (3.5)	92.2 (2.4)	75.0 (5.0)	84.7 (3.5)	78.3 (5.2)	84.7 (3.7)	76.5 (6.1)	82.2 (4.3)	85.0 (6.0)	77.4 (4.1)	0.5393
Social Functioning	73.5 (6.8)	70.0 (4.7)	59.6 (6.9)	79.8 (4.8)	70.4 (7.0)	82.1 (4.8)	74.0 (8.5)	70.7 (5.9)	89.1 (6.7)	70.6 (4.4)	0.7095
Global health status/QoL	54.1 (5.3)	55.3 (3.8)	57.7 (5.6)	68.2 (3.9)	73.0 (5.0)	65.7 (3.6)	72.7 (7.2)	63.1 (5.1)	64.8 (6.8)	68.0 (4.6)	0.8857
Fatigue	32.0 (5.9)	39.0 (4.2)	47.0 (7.0)	36.1 (4.8)	35.1 (6.7)	41.4 (4.7)	30.3 (7.4)	38.8 (5.2)	35.7 (7.2)	39.7 (4.9)	0.7902
Nausea/Vomiting	11.1 (3.4)	6.3 (2.4)	11.5 (3.1)	4.9 (2.2)	11.7 (4.6)	8.1 (3.2)	10.0 (4.0)	5.5 (2.8)	8.3 (6.9)	14.7 (4.5)	0.6123
Pain	27.3 (7.1)	38.8 (5.1)	33.7 (6.8)	24.2 (4.7)	25.7 (6.3)	24.5 (4.4)	29.3 (7.6)	28.2 (5.3)	25.5 (7.9)	30.4 (5.3)	0.8112
Dyspnoea	13.9 (5.4)	19.1 (3.9)	34.3 (6.4)	19.3 (4.4)	30.0 (7.1)	22.5 (4.9)	31.1 (10.3)	38.0 (7.2)	26.9 (8.3)	32.5 (5.6)	0.7507
Insomnia	43.4 (6.6)	34.3 (4.7)	42.4 (7.2)	23.6 (4.9)	35.0 (7.4)	29.7 (5.2)	32.9 (8.3)	30.1 (5.8)	37.3 (8.6)	28.4 (5.7)	0.2236
Appetite loss	23.1 (6.6)	21.6 (4.7)	21.2 (7.4)	15.5 (4.9)	19.2 (6.4)	16.6 (4.5)	14.6 (5.9)	14.1 (4.1)	16.9 (7.1)	17.7 (4.6)	0.7290
Constipation	12.8 (6.1)	23.3 (4.2)	28.5 (7.8)	19.6 (5.4)	22.5 (6.9)	20.2 (4.8)	28.3 (7.5)	11.3 (5.2)	12.3 (9.2)	24.0 (6.2)	0.5646
Diarrhoea	12.6 (3.8)	3.3 (2.6)	23.7 (5.3)	1.9 (3.7)	17.6 (4.3)	4.3 (2.9)	23.1 (5.7)	3.6 (3.9)	9.3 (3.9)	4.5 (2.7)	**0.0014**
Financial Problems	9.7 (5.3)	14.0 (3.7)	27.2 (6.7)	16.0 (4.6)	29.3 (7.2)	19.3 (4.9)	25.2 (7.7)	25.7 (5.5)	19.0 (6.2)	18.9 (4.2)	0.4670
Body image	80.9 (4.5)	84.5 (3.0)	63.7 (6.4)	80.4 (4.5)	74.7 (5.1)	86.0 (3.4)	70.3 (6.3)	83.4 (4.3)	71.1 (6.8)	76.2 (4.5)	0.0570
Sexual functioning	19.6 (5.6)	14.2 (3.6)	17.9 (5.5)	16.9 (3.8)	33.6 (6.6)	13.9 (4.6)	28.7 (6.9)	16.5 (4.9)	25.6 (7.4)	15.7 (4.9)	0.1086
Future perspective	29.1 (6.9)	36.6 (4.7)	27.2 (7.2)	44.0 (5.2)	45.5 (8.0)	49.8 (5.4)	34.9 (9.1)	45.2 (6.3)	59.6 (10.2)	50.9 (6.6)	0.4525
Systemic therapy	16.7 (3.5)	18.0 (2.4)	45.3 (4.4)	22.7 (3.1)	19.5 (3.7)	19.3 (2.6)	23.8 (4.9)	24.7 (3.3)	20.2 (4.1)	22.8 (2.8)	0.2588
Breast symptoms	18.2 (4.5)	20.9 (2.9)	16.5 (4.0)	13.6 (2.7)	13.6 (3.2)	10.9 (2.2)	8.7 (4.7)	12.8 (3.2)	8.4 (4.9)	9.5 (3.2)	0.9740
Arm symptoms	14.0 (4.0)	17.0 (2.6)	16.4 (5.4)	23.6 (3.7)	14.3 (4.9)	21.7 (3.4)	18.2 (6.2)	26.7 (4.3)	9.9 (5.9)	20.6 (3.9)	0.1347
Hair loss	3.9 (3.1)	3.2 (2.2)	43.7 (8.5)	23.6 (5.9)	0.2 (6.0)	9.9 (3.9)	10.2 (6.8)	14.0 (4.6)	21.4 (9.8)	7.9 (6.2)	0.4334

*Legends: QoL. Quality of life*

^*a*^*Estimates for themean scores estimated via the linearmixedmodeling expressed in absolute score points of the scale. Higher values for the symptomscales (Diarrhea, Loss of appetite, Nausea/vomiting, Fatigue) represent aworse level of symptoms. Higher values for the global health/Quality of Life scale represents a better level of functioning*

^*b*^*p-value belongs to the comparison between first line chemotherapy and first line any other therapy*

#### Qol by site of metastases

Twenty-nine women with bone metastases only and 46 women with visceral ±bone metastases completed QoL assessments at baseline. Interestingly, women with bone metastases only reported worse physical functioning (59.8 vs 77.9; *p*=0.0079) and role functioning (55.9 vs 74.8; *p*=0.0412) on the functional scales of the QLQ-C30, as well as more pain (mean 52.0 vs 24.6; *p*=0.0066) compared to women with visceral ± bone metastases. All differences were statistically significant and clinical relevant. Differences at baseline were not visible anymore until the last visit at 24 months (Table 8).

**Table 8:** QoL assessement (EORTC QLQ C30 and EORTC QLQ BR 23) by site of metastases and time (Mean scores and standard errors)

	*Baseline*		*6 Mo*		*12 Mo*		*18 Mo*		*24 Mo*		
*QOL domain*	*bone only*	*visceral +/- bone*	*bone only*	*visceral +/- bone*	*bone only*	*visceral +/- bone*	*bone only*	*visceral +/- bone*	*bone only*	*visceral +/- bone*	*p-Value*^*b*^
Physical Functioning	59.8 (5.0)	77.9 (3.9)	74.3 (5.6)	73.1 (4.2)	68.6 (5.5)	73.4 (4.2)	67.9 (6.7)	67.9 (5.3)	60.0 (6.2)	71.1 (5.2)	0.5868
Role Functioning	55.9 (6.5)	74.8 (5.2)	62.0 (7.6)	65.1 (5.6)	65.0 (6.8)	67.8 (5.3)	67.1 (8.4)	62.6 (6.9)	61.7 (9.2)	63.9 (7.8)	0.9142
Emotional Functioning	58.1 (4.8)	61.6 (3.8)	70.2 (6.1)	65.7 (4.4)	68.9 (5.1)	72.2 (4.0)	66.9 (6.1)	67.9 (4.9)	63.8 (6.0)	75.2 (5.2)	0.6476
Cognitive Functioning	89.4 (3.2)	91.1 (2.5)	82.3 (4.8)	81.2 (3.6)	83.1 (4.9)	81.7 (3.9)	79.5 (5.7)	80.5 (4.5)	77.4 (5.4)	81.3 (4.7)	0.9127
Social Functioning	69.3 (6.3)	72.8 (5.0)	81.0 (6.9)	70.0 (5.0)	77.7 (6.4)	77.7 (5.1)	69.5 (7.7)	72.4 (6.4)	65.3 (5.5)	84.5 (4.7)	0.6739
Global health status/QoL	53.2 (5.0)	55.3 (3.9)	65.0 (5.7)	65.6 (4.0)	65.5 (4.8)	69.5 (3.8)	60.8 (6.6)	70.0 (5.5)	57.4 (5.3)	74.3 (4.8)	0.1450
Fatigue	43.9 (5.5)	32.2 (4.3)	40.9 (6.9)	37.8 (5.0)	45.1 (6.2)	36.1 (4.9)	37.7 (6.9)	35.4 (5.6)	41.5 (6.3)	36.7 (5.3)	0.5008
Nausea/Vomiting	7.6 (3.2)	8.1 (2.5)	3.1 (3.1)	8.7 (2.3)	13.6 (4.2)	5.6 (3.3)	7.7 (3.6)	6.7 (2.9)	15.8 (5.2)	4.8 (4.4)	0.2928
Pain	52.0 (6.4)	24.6 (5.0)	28.3 (6.7)	25.7 (4.9)	29.1 (5.7)	22.4 (4.5)	31.7 (7.0)	27.0 (5.6)	38.6 (6.7)	21.9 (5.5)	0.2660
Dyspnoea	22.3 (5.2)	14.4 (4.0)	24.7 (6.4)	22.5 (4.6)	25.0 (6.6)	25.8 (5.2)	44.4 (9.4)	30.1 (7.6)	38.4 (7.5)	26.1 (6.1)	0.3687
Insomnia	33.3 (6.1)	40.1 (4.8)	19.1 (7.1)	34.4 (5.0)	28.0 (6.9)	34.0 (5.4)	28.9 (7.4)	32.3 (6.1)	35.0 (7.1)	26.8 (6.1)	0.5571
Appetite loss	22.2 (6.2)	22.1 (4.9)	16.1 (7.0)	17.8 (5.1)	19.0 (6.0)	16.6 (4.7)	12.9 (5.3)	15.5 (4.4)	22.9 (5.4)	11.5 (4.8)	0.6599
Constipation	22.9 (5.6)	17.9 (4.5)	22.4 (7.6)	23.2 (5.6)	17.1 (6.4)	23.7 (5.0)	12.3 (7.0)	20.0 (5.6)	28.1 (7.3)	12.4 (6.4)	0.9843
Diarhoea	3.3 (3.6)	8.3 (2.8)	4.2 (5.5)	12.6 (4.1)	9.6 (4.1)	8.5 (3.2)	8.1 (5.5)	11.5 (4.4)	7.1 (3.5)	5.3 (2.9)	0.6297
Financial Problems	7.6 (4.9)	15.5 (3.8)	9.9 (6.5)	24.2 (4.7)	20.6 (6.6)	24.0 (5.2)	23.8 (7.1)	26.6 (5.6)	18.7 (5.4)	18.8 (4.5)	0.4584
Body image	81.8 (4.0)	84.1 (3.3)	80.4 (6.3)	72.1 (4.7)	83.7 (4.6)	81.8 (3.6)	74.5 (5.7)	82.0 (4.6)	68.1 (5.9)	78.1 (4.8)	0.7569
Sexual functioning	15.7 (4.8)	15.9 (4.0)	17.5 (5.2)	17.5 (3.9)	11.4 (6.1)	26.0 (4.9)	13.7 (6.2)	24.9 (5.2)	14.0 (6.2)	22.2 (5.4)	0.1794
Future perspective	33.2 (6.2)	35.5 (5.1)	42.6 (7.3)	36.3 (5.3)	47.2 (7.3)	48.1 (5.7)	45.8 (8.3)	38.7 (6.7)	37.1 (7.7)	66.8 (6.7)	0.5522
Systematic therapy	21.8 (3.1)	14.9 (2.5)	26.1 (4.9)	31.6 (3.6)	22.9 (3.3)	17.5 (2.6)	26.9 (4.4)	23.2 (3.5)	26.0 (3.6)	20.3 (3.0)	0.5631
Breast symptoms	22.4 (3.8)	18.8 (3.2)	15.4 (3.8)	13.9 (2.8)	13.0 (3.0)	11.2 (2.3)	10.1 (4.3)	12.4 (3.4)	13.9 (3.9)	5.3 (3.4)	0.4986
Arm symptoms	21.4 (3.4)	12.6 (2.7)	24.4 (5.2)	19.4 (3.9)	22.3 (4.6)	17.4 (3.6)	29.4 (5.6)	20.7 (4.4)	21.0 (5.1)	14.5 (4.2)	0.2467
Hair loss	7.2 (2.7)	1.2 (2.2)	29.7 (8.5)	30.0 (6.3)	16.2 (5.3)	2.6 (3.9)	23.8 (5.9)	7.1 (4.6)	15.0 (7.9)	9.7 (6.5)	0.1468

*Legends: QoL: Quality of life*

^*a*^*Estimates for themean scores estimated via the linearmixedmodeling expressed in absolute score points of the scale. Higher values for the symptomscales (Diarrhea, Loss of appetite, Nausea/vomiting, Fatigue) represent aworse level of symptoms. Higher values for the global health/Quality of Life scale represents a better level of functioning*

^*b*^*p-value belongs to the comparison between metastase location groups*

## Discussion

Treatment of women with MBC aims to prolong survival and improve or maintain QoL [4]. Our results indicate that primary surgery does not appear to improve QoL in patients presenting with MBC. QoL assessments in these women are critical and many phase 3 trials in this population include QoL as a primary or secondary endpoint [18]. The ABCSG 28 is the third randomised trial evaluating the role of primary surgery in women with stage IV BC, but the first to report the impact of primary surgery prior to systemic therapy versus primary systemic therapy on QoL [13]. Two previous randomised trials [7, 12] of surgery vs. no surgery described oncologic outcomes; QoL data from one of these trials have been presented in abstract form [19].

Our trial, which was halted prematurely, indicated that primary surgery does not improve OS, TTPd or TTPl in women presenting with MBC [13]. This makes QoL outcomes all the more important. Our results indicate that global health status, physical functioning, social functioning, and future perspective were predictors for OS and/or TTTd. QoL outcomes as predictors for OS in BC have already previously been described in early BC, with the scale future perspective also being a predictor for OS in that setting [20]. This indicates that QoL results, especially the robust scale global health/QoL and future perspective, could be used as an additional marker for prediction of OS and TTTd.

The mean baseline global health/QoL score (54.7±26.1) of patients in our study is in line with reference values (60.2 ± SD 25.5) for recurrent/ metastatic BC [21]. Although patients in the non-surgery arm reported a higher mean score on the Global Health Score/QoL at baseline, this difference disappeared at the first follow-up visit at 6 months after randomization and did not reappear later. The difference at baseline was caused by a rather low score in the surgery arm, while the score in the nonsurgery arm was in line with reference data and other studies [21, 22]. These differences at baseline could be the result of the relatively small number of patients in the surgical arm who completed QoL assessment at baseline. Assessment at the following time point showed no differences between the arms, similar to the results reported by Rajendra et al [22]. On the other hand, the difference may reflect a short term impact of the surgery on QoL and global health score.

Patients in both arms of our study showed clinically significant improvements on the global health/QoL scale as well as on the functional scales emotional functioning and future perspectives. Emotional symptoms in MBC patients are associated with physical symptoms such as pain, insomnia and fatigue and improvement of emotional functioning is clearly important [20, 23–28]. In our trial, insomnia and fatigue were the most severe symptoms at baseline in both arms and remain unchanged over the time.

Patients without surgery reported clinically relevant fewer breast and arm symptoms at the 6 months, indicating that local surgery causes symptoms and morbidity that persist for at least at 6 months. Patients without surgery reported better cognitive function than those with surgery, and the score on the cognitive functioning scale was stable from baseline to the 24-month follow-up. In contrast, in the surgery group cognitive function score decreased over time by more than 10 points, indicating clinically significant worsening. The reason for this is unclear. Hermelink et al [29]. described cognitive impairment in BC patients depending on therapy [chemotherapy vs no chemotherapy] and this was intertwined with posttraumatic syndrome after receiving the diagnosis [29]. Sato et al. looked at the impact of BC surgery on cognitive function and found alterations in brain structure shortly after surgery, particularly in the thalamus, which may be associated with attentional dysfunction [30]. It may however be far-fetched to relate our observation to the immediate effects of the surgical procedure and or anaesthesia.

Analyses of QoL according to age [<60y vs ≥60y] showed that younger women had a higher score on the sexual functioning scale as well as on the physical functioning scale. These results are as expected.

Patients receiving chemotherapy as first-line therapy reported better physical functioning at baseline than patients receiving other systemic treatment. It is however likely that a good baseline performance status in these patients contributes to the selection of patients and decision for chemotherapy.

Interestingly, patients with only bone metastases reported worse physical and role functioning and pain compared to patients with visceral metastases. Pain is a leading symptom in patients with bone metastases, and an important factor influencing QoL [31].

Strengths of our study are the prospective randomized design, good compliance of the patients with QoL assessment, and relatively long follow-up. Apart from one study in abstract form [19], this is the first full publication to evaluate the impact of primary surgery on QoL in patients presenting with MBC.

### Study limitations

A limitation of our study is that it stopped prematurely at 4 years because of slow recruitment. Our findings based on the relatively small number of patients in both arms need to be confirmed in following studies.

## Conclusion

Our prospective randomized trial showed that primary surgery does not improve nor alter QoL of patients with de novo stage IV BC. Global health status and physical functioning were predictors for OS and could be use as additional marker for prediction of OS and TTTd in patients with de novo Stage IV BC.

## Data Availability

The datasets used and analysed during the current study are available from the corresponding author on reasonable request.

## Abbreviations

ABCSG: Austrian Breast and Colorectal Study Group
BC: Breast cancer
CTX: Chemotherapy
EORTC QLQ-BR23: European Organisation for Research and Treatment of Cancer Quality of Life Questionnaire – BR (Breast) 23
EORTC QLQ-C30 core questionnaire: European Organisation for Research and Treatment of Cancer Quality of Life Questionnaire – C30
HR: Hazard ratio
MBC: Metastatic breast cancer
OS: Overall survival
TTPd: Time to distant metastases
TTPl: Time to locoregional metastases
QoL: Quality of life
